# Visualization of relative cochlear motions using high resolution optical coherence microscopy

**DOI:** 10.1016/j.heares.2025.109311

**Published:** 2025-05-17

**Authors:** Scott Page, Roozbeh Ghaffari, Dennis M. Freeman

**Affiliations:** a Research Laboratory of Electronics, Massachusetts Institute of Technology, Cambridge, MA, USA; b Department of Electrical Engineering and Computer Science, Massachusetts Institute of Technology, Cambridge, MA, USA

**Keywords:** Time-domain, Spectral domain, Cochlea, Gerbil Cochlea, Mammalian Cochlea, Relative motion, Differential motion, Cochlea movies, Organ of Corti animation

## Abstract

Despite enormous progress in understanding the electro-mechanical properties of outer hair cells and the molecular basis of these properties, less is known about the relative motion of the organ of Corti and accessory structures that shape cochlear responses to acoustic stimulation. Here, we characterize absolute and relative motions of apical regions of the excised gerbil cochleae using a custom Doppler optical coherence microscopy (DOCM) system. Responses to sinusoidal stimuli show nanometer-scale motions of the tectorial membrane (TM), organ of Corti structures (e.g. outer hair cells, pillar cells), and basilar membrane in the apical turn of the cochlea. Motion-magnified analysis reveals rotations about the inner pillar cells at nearly constant phase, whereas TM motion lags that of the underlying cells by as much as 0.1 radians. Our DOCM results demonstrate a new technique capable of concurrent high resolution anatomical imaging and nanometer-scale motion analysis of cellular and acellular structures in response to stapes stimulation, enabling investigations of relative cochlear motions and feedback mechanisms.

## 1. Introduction

Motion measurements have confirmed that high sensitivity, sharp tuning, and nonlinearity emerge in the mechanical stages of auditory processing (Brownell et al, 1985; Robles and Ruggero, 2001; Ashmore, 2008). It is now widely accepted that these properties ultimately derive from active mechanical amplification, although there is a paucity of direct *in situ* or *in vivo* results describing the hydromechanical pathways that underlie this amplification. Much of our understanding of cochlear mechanisms is largely based on measurements from the base of the cochlea. The basilar membrane has typically been approached near the base via direct optical access through the round window membrane (or via perforation of the otic capsule) using Mossbauer (Johnstone and Boyle, 1967), laser Doppler vibrometry (Cooper and Rhode, 1992, 1995; Recio et al, 1998) and most recently, optical coherence techniques (see references in Olson et al, 2024).

Recent investigations have uncovered new modes of motions that appear to exist in the apical turns of the cochlea, the region most critical for speech, and disappear at high frequencies (Nowotny and Gummer, 2006; Guinan, 2013; Lichtenhan et al, 2014). These modes of motion cannot be explained by excitation via the basilar membrane wave (Guinan and Cooper, 2008). Understanding these new modes of motion could prove to be invaluable for our understanding of cochlear feedback mechanisms and interactions across acellular and cellular sub-structures within the cochlear partition.

To address these challenges, we have developed a Doppler optical coherence microscopy (DOCM) system (Page, 2015) that measures nanometer-scale motions. The DOCM system combines the principles of laser Doppler vibrometry (LDV) with optical coherence tomography (OCT), whereby DOCM images are constructed using optical range information, and high-resolution images can be acquired. As with LDV, the DOCM system uses a heterodyne method to shift information about audio frequency motion to higher frequencies, where motions can be detected without contamination by low-frequency noise and without phase ambiguity. This combination of LDV and OCT is used to measure nanometer-scale displacements of weakly scattering structures throughout the cochlear partition.

The goal of this study is to visualize, with high lateral resolution, axial displacements of the low frequency apical region in the excised *in situ* (excised, yet cochlear turns intact) cochlear partition in response to stapes stimulation.

## 2. Methodology

### 2.1. Animal preparation and alignment

**Fig. 1.**
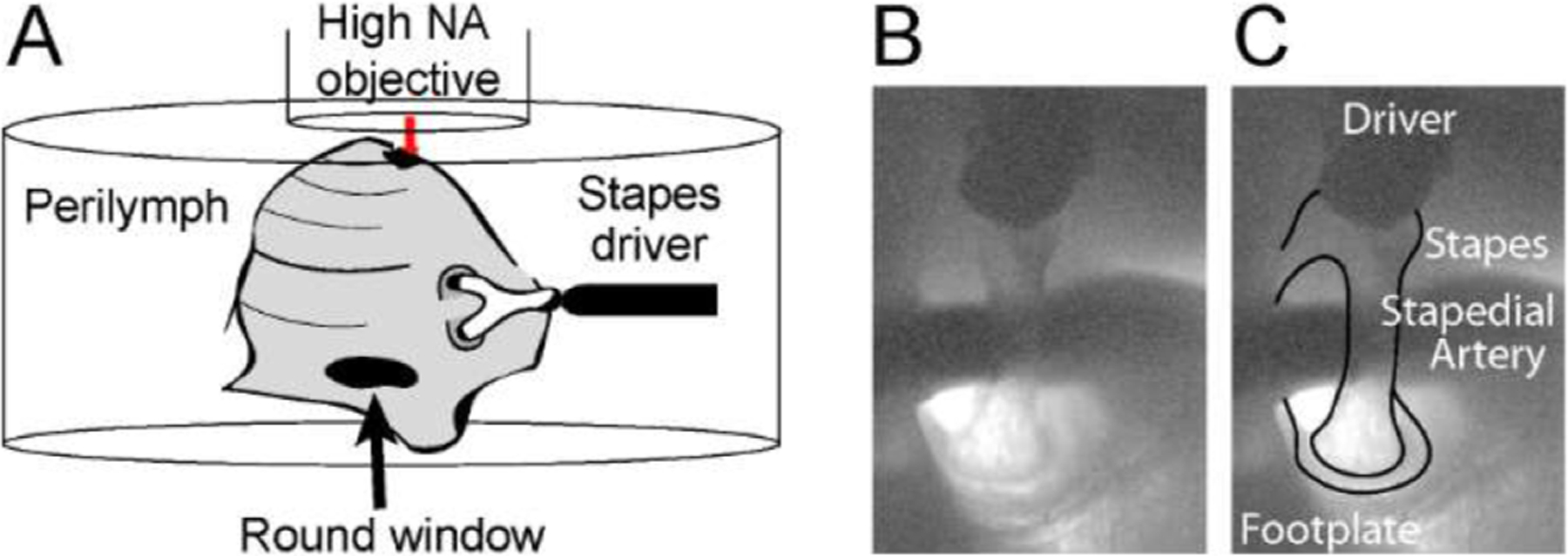
*In situ* preparation. (A) Stimulation and imaging. Cochleae are affixed using dental cement to a Petri dish. Cochlear motions were stimulated by vibrating the stapes with a titanium probe attached to a piezoelectric crystal. The resulting motions are imaged using a high numerical aperture objective with optical access through an approximate 0.5 mm^2^ apical hole. (B, C) Close-up image and labeled view of titanium probe in contact with the stapes.

Mongolian gerbils age 3–5 weeks of either sex were euthanized via carbon dioxide asphyxiation and decapitated. The bulla containing the cochlea was isolated and opened. Care was taken to preserve the stapes so that the cochlea could be stimulated by vibrating the stapes. The stapedius and tensor tympani tendons were precisely cut to avoid stapes damage upon incudo-stapedial joint and middle ear separation. Isolated cochleae were affixed to a Petri dish using two-part dental cement (Durelon, ESPE Dental-Medizin GmbH) and bathed in an artificial perilymph solution with low calcium and chloride content to reduce hair cell blebbing (Aranyosi and Freeman, 1999; Aranyosi, 2002) (Fig. 1). The pH of artificial perilymph was maintained at 7.30 to approximate biological pH and consisted of 7 mM sodium chloride (NaCl), 163.4 mM sodium gluconate (C_6_H_11_NaO_7_), 3 mM potassium chloride (KCl), 0.1 mM calcium chloride dihydrate (CaCl_2_⋅2H_2_O), 0.1 mM magnesium chloride (MgCl_2_), 2 mM sodium sulfate (Na_2_SO_4_), 0.5 mM sodium dihydrogen phosphate (NaH_2_PO_4_), 5 mM HEPES (C_8_H_18_N_2_O_4_S), 5 mM dextrose (C_6_H_12_O_6_), and 4 mM L-glutamine (H_2_NCOCH_2_CH_2_ CH(NH_2_) CO_2_H).

Fig. 1A diagrams the apex of a gerbil cochlea immersed in artificial perilymph and positioned under a high numerical aperture (0.8 NA) water immersion objective. As our goal was high resolution motion measurements of the cochlear partition, a high NA objective allowed for efficient light collection from even weakly scattering structures, such as the tectorial membrane, at high lateral resolution. Resolution and light collection were further improved by gently shaving a small hole (approximately 0.5 mm^2^) with a #11 scalpel blade in the apical part of the temporal bone. Due to the short working distance of the high NA objective, the small hole remained open throughout our measurements.

Visible light from a dichroic mirror allowed angular alignment of the apical turn and setting the programmed measurement beam path. As interferometric measurements are sensitive only to displacements along the optical axis, interpretation of these measurements can be complicated when the important structures are not perpendicular to the measurement axis. To reduce the effects of skew (Frost et al, 2022; Olson et al, 2024) and to focus on axial motions of the basilar membrane, reticular lamina, and tectorial membrane, we adjusted the tilt of the preparation to minimize the difference between the axis of outer hair cells and the optical axis of our measurement system.

A piezoelectric driver coupled to a titanium probe was mounted to a micromanipulator and used to deliver sinusoidal mechanical stimuli to the stapes. The tip of the probe was coated with dental cement and brought into gentle contact with the stapes as shown in Fig. 1B, C. Pre-calibrated frequency dependent voltages were applied to the piezo such that the stapes induced piston-like constant equivalent stapes-induced sound pressure level across frequency (< 1kHz). The characteristic frequency of the cochlear partition measurement site was determined by measuring displacements of a small region of the reticular lamina as a function of frequency.

Forty-two gerbils were used in the development of the animal preparation and the imaging and motion measurement methods. We obtained good image optical alignment, high resolution images, and nanometer-scale motion measurements in four of the 42 preparations. From these four, we chose one that is representative, and all of the results shown in this manuscript are from that preparation. The displacement of the reticular lamina peaked at 275 Hz in this preparation, and all subsequent measurements were done at that frequency with a peak stapes displacement of 20 nm, which corresponds to an approximate equivalent sound pressure of 87 dB SPL (Rosowski et al, 1999; Ravicz et al, 2008). Preparation time was approximately 20 minutes after euthanasia. The care and use of animals in this study were approved by the Massachusetts Institute of Technology Committee on Animal Care.

### 2.2. Optical system

Our time-domain DOCM system is constructed using custom optics (Hong and Freeman, 2006; Page et al., 2015) and is based on a Michelson interferometer in a double-pass configuration. Spatially coherent broadband light, generated by a single source superluminescent diode (SLD: center wavelength, 843 nm; full width at half maximum FWHM bandwidth, 52.6 nm), is polarized via two quarter wave plates and a half wave plate. A polarization insensitive optical isolator prevents backscattered light from entering the SLD. The output of the SLD is split into two paths: the sample path, which is focused onto the cochlea via an objective lens; and the reference path, which is directed onto a retroreflector at the desired measurement depth. The optical paths are upshifted in frequency by 80 MHz and 80.25 MHz, respectively, on the way to and from their destinations via acousto-optic modulator. This effectively upshifts the interferometric signal to a difference frequency of 500 kHz, bringing it out of the baseband noise. Due to the low coherence of the source light, interference occurs only when the sample and reference path lengths are matched to within the coherence gate (~6.5 μm).

**Fig. 2.**
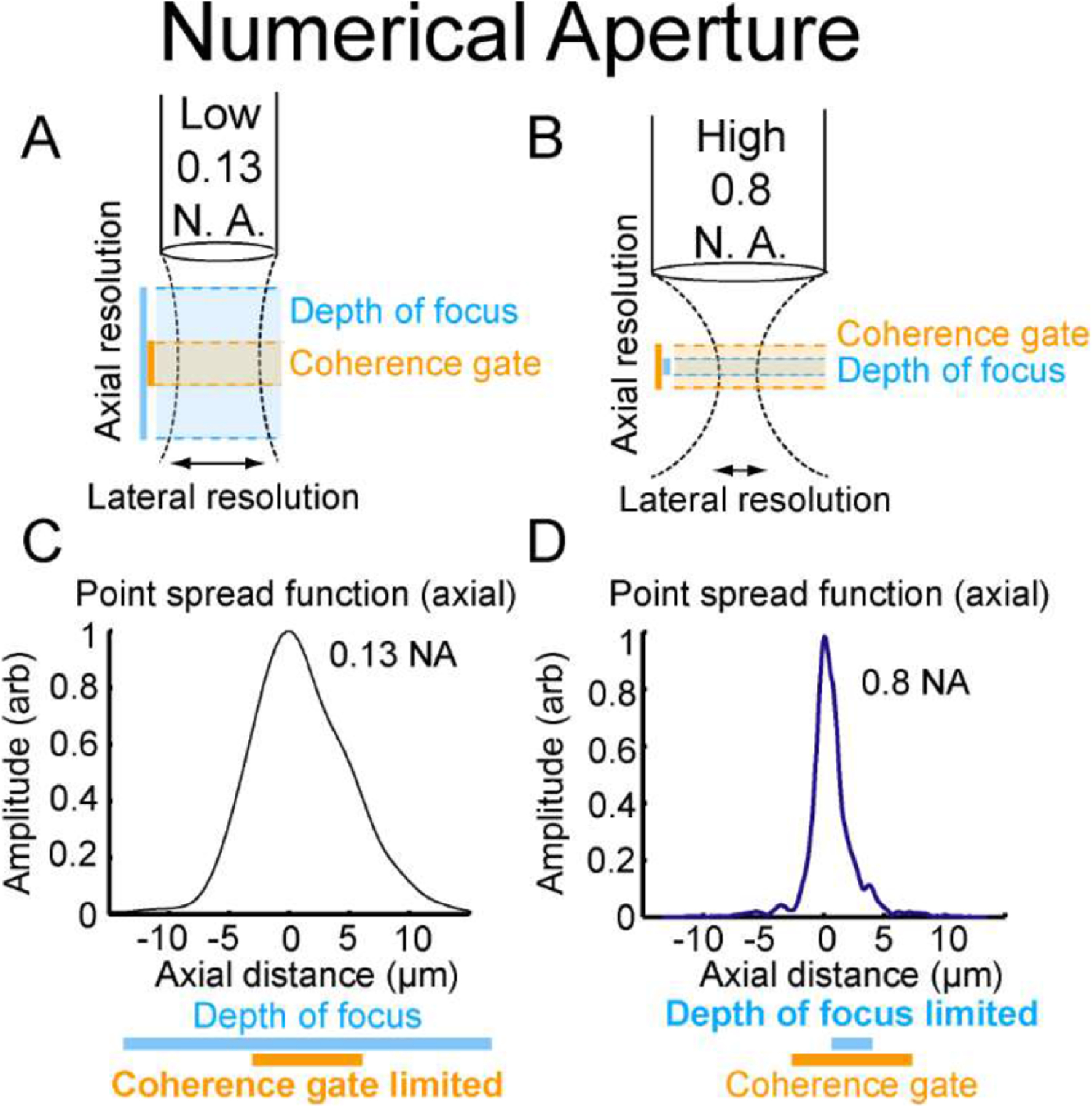
Schematic diagram of optical setup with (A) low NA objective and (B) High NA objective highlighting differences in depth of focus (blue), coherence gate (orange), and lateral resolution (horizontal arrows). Axial point spread functions for our system with (C) a low NA objective, which is limited by coherence gate (orange), and (D) a high NA objective, which is limited by depth of focus (blue).

Axial resolution is determined by the smaller of either the coherence gate or depth of focus of the objective, while lateral resolution is set by the source light wavelength and objective (Page et al, 2015). Fig. 2 shows the effect of low 0.13 NA (Fig. 2A, C) and high 0.8 NA (Fig. 2B, D) objectives on axial resolution in our system. Axial resolution with the low NA objective is limited by the coherence gate (~6.5 μm), whereas that of the high NA objective is limited by the depth of focus of the objective (~2.4 μm). The lateral resolution of a 0.13 NA objective is ~2.4 μm, while that for a 0.8 NA objective is ~ 340 nm.

Axial scanning was accomplished by moving the sample objective lens and reference retroreflector together on a piezoelectric scanning stage (Physik Instruments), while lateral scanning was accomplished using linear stepper motor stages. Isolated cochleae were placed under a 40x water immersion objective on a lateral-axes motor driven scanning stage. The objective was mounted to a piezoelectric driven axial stage. Together, these stages allowed for axial motions to be measured at each point throughout the isolated preparation. Cross-sectional scans were acquired by first moving the piezoelectric driven axial stage (A-scan), followed by an increment in the lateral motor stages (B-scan), then repeating the process. The data presented in this study were acquired within an hour of euthanasia.

### 2.3. Data acquisition

**Fig. 3.**
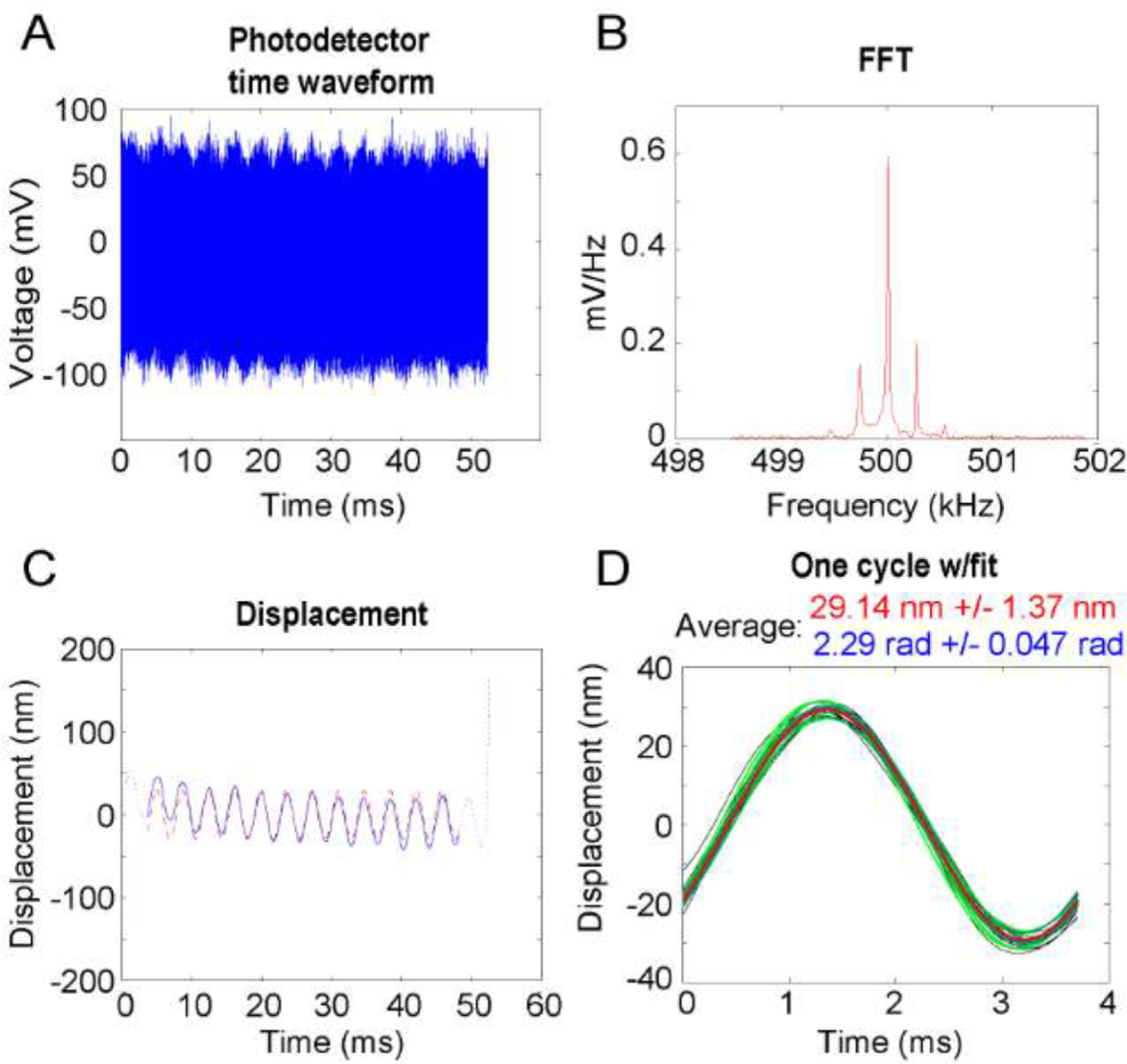
DOCM interferometric signal processing in the *in situ* Mongolian gerbil (G8208) with ~87 dB equivalent sound pressure level via sinusoidal stapes stimulation at 275 Hz. (A) Photodetector interferometric signal acquired from 1 point. (B) Spectral content of interferometric signal. (C) Axial displacement (solid blue line) and fitted sinusoid (red dashed line) after Hilbert transform demodulation and signal processing. (D) Comparison of curve fitting across cycles.

Light from the superluminescent diode was focused to illuminate a single point in the target, and backscattered light was detected with a photodiode. The 500 kHz signal acquired by the photodetector was sampled at a rate greater than the Nyquist rate (Fig. 3A, B). The amount of time spent at each pixel is a function of the amount of averaging desired. In the case of pure-tones, it is a function of the frequency of the motion and the number of cycles desired for averaging. Based on measurements of known piezoelectric motion, we decided to average at least 10 cycles and discard the first and last cycles to reduce interpixel transients. Displacement is determined by extracting the difference in instantaneous phase of the interferometric signal from that of the acousto-optic modulation reference signal using a Hilbert transform (solid blue line in Fig. 3C). In this case, 14 cycles of 275 Hz displacement were acquired, of which 12 cycles (first and last excluded) were used to fit a least squares temporary estimate of amplitude and phase of the motion (dotted red line in Fig. 3D). By breaking up the measured displacement signal into its constituent cycles, and comparing each to the least squares estimate, the error of the amplitude and phase of each cycle can be extracted. From these, the median amplitude and phase are chosen to represent the motion of that pixel, and the standard deviation of phase over all cycles represent the confidence of that motion. This approach of oversampling cycles and selecting the median value amplitude and phase, greatly reduces the effect of outliers in the motion data.

### 2.4. Animated visualizations

To visualize the cochlear motions that result for a given frequency of excitation, we would like to view our interferometric results as an animation. However, the nanometer-scale displacements of cochlear structures are so small that the measured motions would barely blur the micrometer-size structures. To overcome this difficulty, we magnify the measured motions by a constant factor to construct a motion-magnified animation that can be viewed at a slow rate (typically tens of hertz) chosen to facilitate human observation.

Motion magnification is based on a “constant brightness” assumption (Horn and Schunck, 1981). The idea is that in-plane motions of a structure do not change the brightness of the target. Rather, such motions simply shift the locations of sources of that brightness. Subpixel motions of an image thereby displaces the pixels that were originally on a Cartesian grid to new non-Cartesian positions. By resampling the displaced image on original pixel locations, we can approximate the changes in pixel brightnesses that would have resulted from the motion. Even more interestingly, we can “magnify” the measured motions by simply scaling the corresponding displacements.

When the magnification factor is high, motion magnification can distort images, especially when the measured motion field is noisy. For that reason, it is important to reduce imaging noise (as discussed above) and limit the magnification factor accordingly. Additionally, it is important to create an effective grouping of neighboring pixels, which do not move independently in real world applications (Liu et al, 2005; Wadhwa et al., 2017). The amplitude and phase of each pixel was recalculated based on a complex average of its surrounding pixels, weighted by their confidence measurement (standard deviation of phase), and their distance from the center pixel. This has the effect of reducing the effect of errant measurements, as well as providing a realistic interpretation of how the cochlear partition moves as a whole.

## 3. Results and discussion

### 3.1. High resolution imaging

**Fig. 4.**
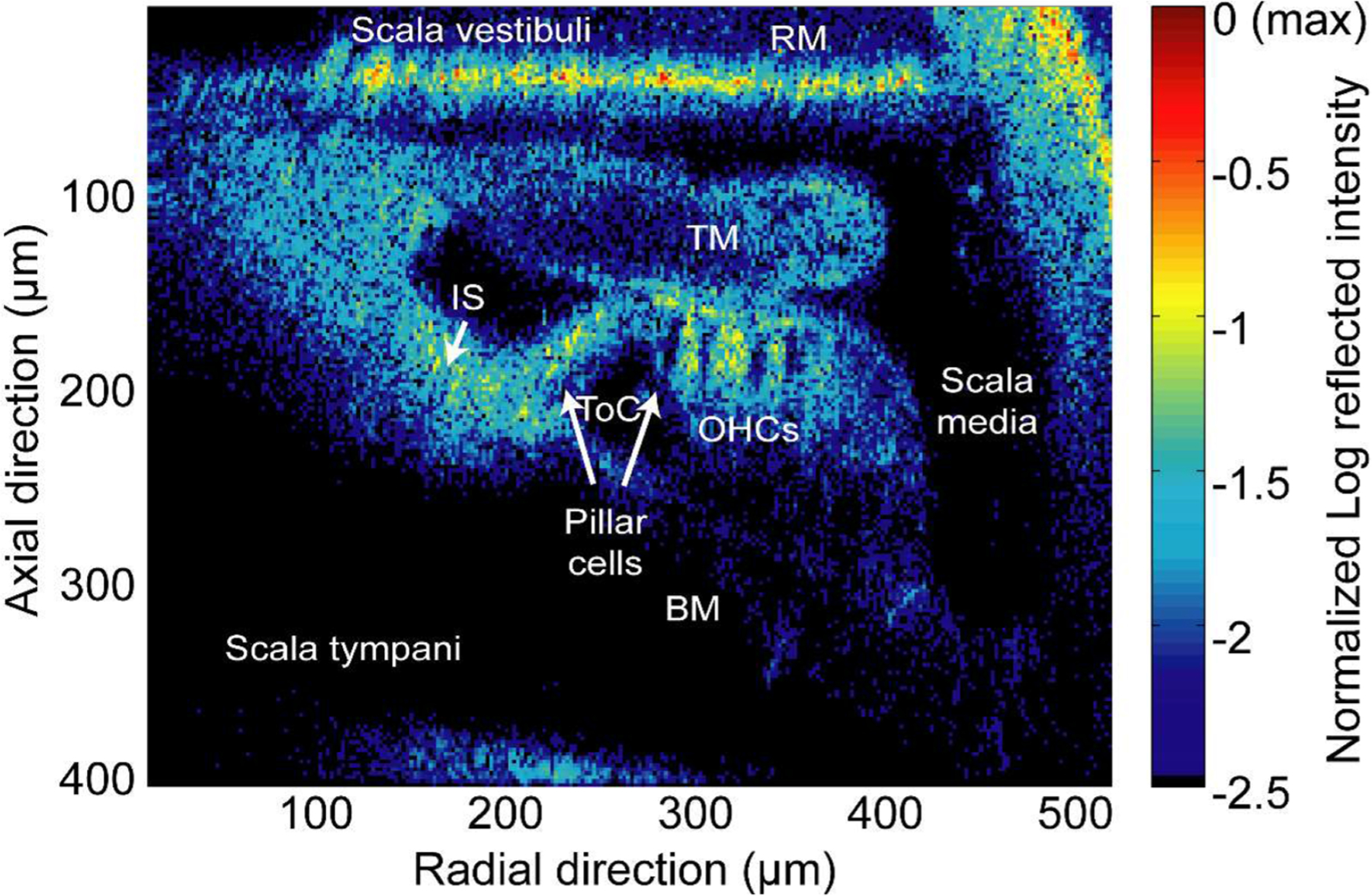
Apical cross-section (275 Hz characteristic place) of an *in situ* Mongolian gerbil (G8208) cochlear partition. Abbreviations: basilar membrane (BM), tunnel of Corti (ToC), inner pillar cell (IPC), outer hair cells (OHCs), tectorial membrane (TM), inner sulcus (IS), and Reissner’s membrane (RM).

Fig. 4 shows a high resolution image of the cochlear partition of an excised *in situ* gerbil cochlea preparation at the approximate 275 Hz characteristic place. Images are normalized to their maximum brightness and then viewed on a base ten logarithmic scale in order to view a large range of reflectivity, and effectively bring into view weakly scattering structures such as the tectorial membrane. Structures such as the basilar membrane (BM), tunnel of Corti (ToC), inner pillar cell, outer hair cells (OHCs), tectorial membrane (TM), inner sulcus, and Reissner’s membrane are clearly visible.

The high spatial resolution achieved by this system results primarily from the use of a high NA (0.8) objective, the optical access hole, and the excised nature of the preparation. Lateral resolution is primarily determined optically in both low and high NA systems, because the coherence gate only affects axial resolution. Increasing NA improves lateral resolution of an optical system, but it comes with costs of decreased working distance and, in the case of our system, longer scanning times as the objective must be repositioned for every pixel since the depth of focus is smaller than the coherence gate.

### 3.2. Motion measurements

**Fig. 5.**
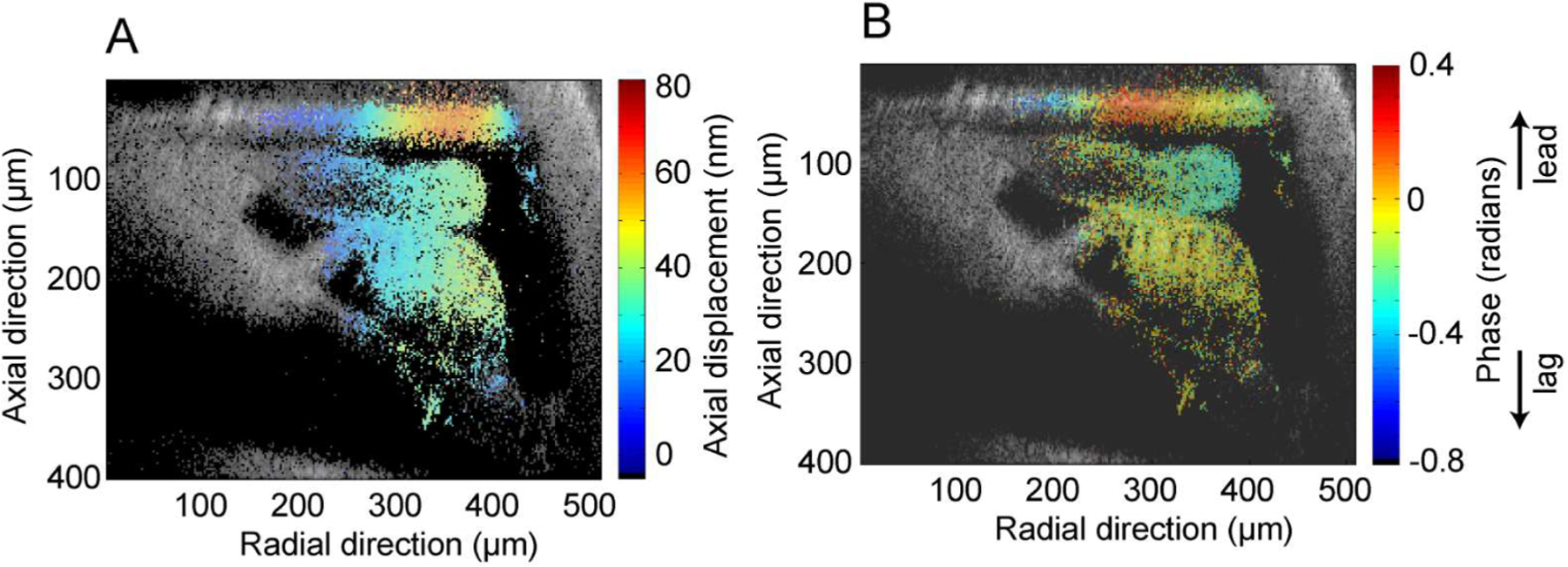
(A) Displacement and (B) Phase of apical cross-section (275 Hz characteristic place) of an *in situ* Mongolian gerbil (G8208) cochlear partition in response to ~87 dB SPL equivalent sound pressure level stapes stimulation at 275 Hz.

Cochlear motions are nanometer in scale, compared to the micrometer scale size of the cochlear structures of interest. Our system provides simultaneous nanometer-scale resolution motion measurements at each spatial point, with the same axial (~2.4 μm) and lateral resolution (~ 390 nm) seen in Fig. 4. These motion measurements come through a separate directionally sensitive heterodyne channel and yield displacements and phases of structures throughout the partition. Fig. 5 shows displacement and phase maps in response to a 275 Hz ~87 dB SPL sinusoidal input at the stapes. The measured motions increase with radial distance from the modiolus, pivoting about the foot of the inner pillar cell. Interestingly, motions are nearly in phase throughout the organ of Corti. However, there is an increasing phase lag of the tectorial membrane as radial distance from the limbal attachment increases, and a significant phase delay in axial motions of the tectorial membrane when compared to the underlying structures and overlying Reissner’s membrane.

### 3.3. Magnified motion animation

Although much of the cochlear partition at a given location along the spiral undergoes motion due to sound-induced pressure waves traveling the length of the cochlea, we are most interested in how these structures interact relative to each other. Structures move with these waves with different magnitudes and phases, interacting with each other in complex ways. One approach to get an overarching view of what is globally occurring, is to magnify the motions of these structures and view “in real-time,” the cross-sectional partition’s response to sound as an animation.

**Fig. 6.**
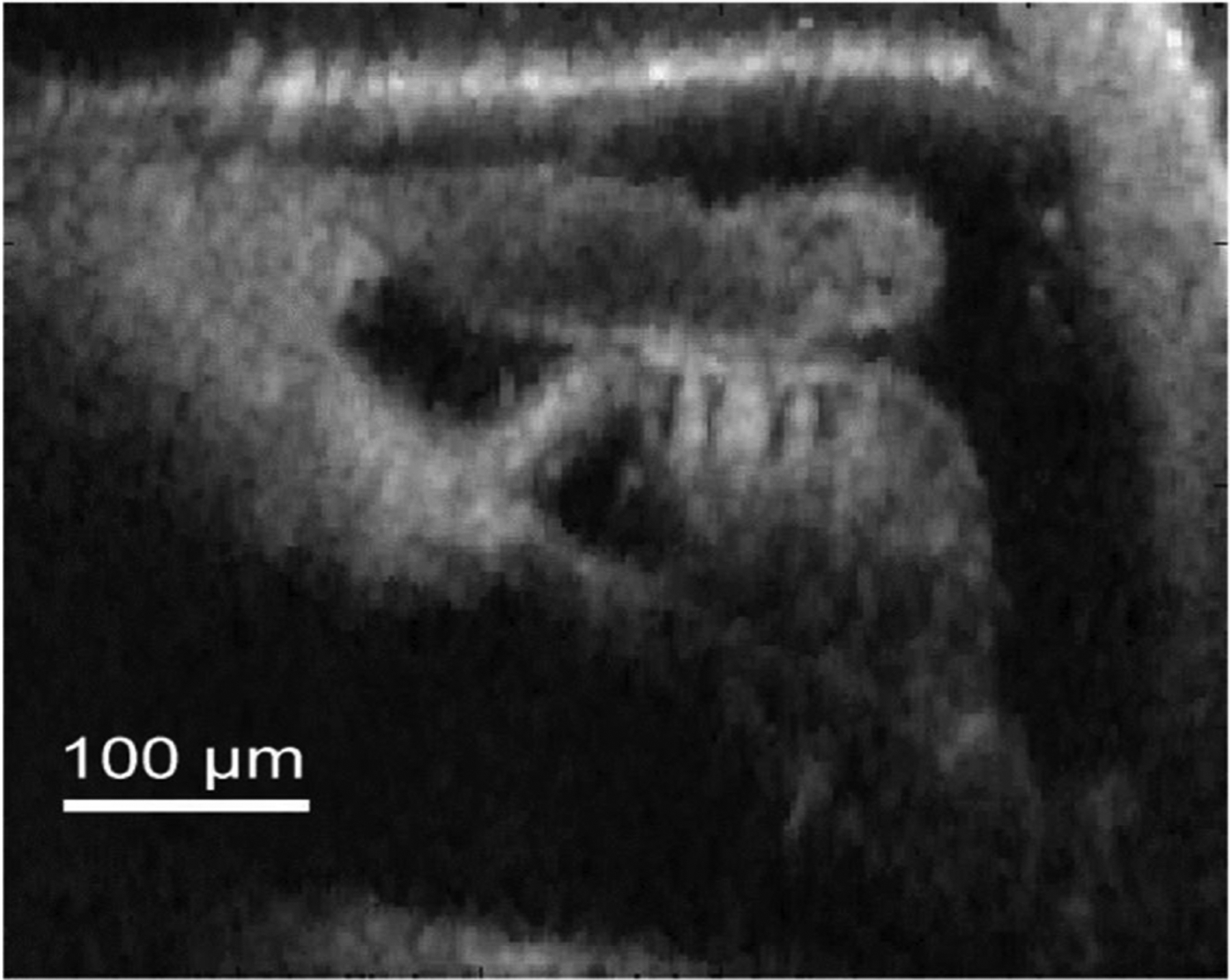
First frame of an animation of 150x amplified axial absolute motion in response to a ~87 dB SPL equivalent stapes sinusoid stimulus. See associated video in Supplementary Materials Section.

Fig. 6 shows the first frame of an animated representation (see associated video in Supplementary Materials Section) of the axial motion of the cochlear partition in response to a 275 Hz ~87 dB SPL equivalent stapes sinusoid stimulus. Although the sound-induced displacements (~10–80 nm) are much smaller than the pixel spacing in this image (2 × 2 μm), the motions are clearly visible because they have been magnified 150 times (see section 2.4). The animation illustrates the rocking motions of the organ of Corti about a pivot near the feet of the inner pillar cells. While we see only slight phase differences within the organ of Corti, we see significant differences in the phase of the tectorial membrane.

### 3.4. Relative motion - a change in frame of reference

As with all OCT systems, our system reports measurements of cochlear motions in an absolute frame of reference relative to the apparatus. However, motions of one structure relative to another can be more useful in assessing cochlear function. For example, the difference in axial displacements of the basilar membrane and reticular lamina can be small compared to the absolute displacements of either structure. To better visualize small relative displacements, we estimate the complex magnitude and phase of measured absolute motion along a reference plane, and then subtract it from the absolute displacements along its physiologically relevant axis. The result of this operation is to stop the motion of the reference structure and view all structures in a frame of reference attached to that reference structure.

**Fig. 7.**
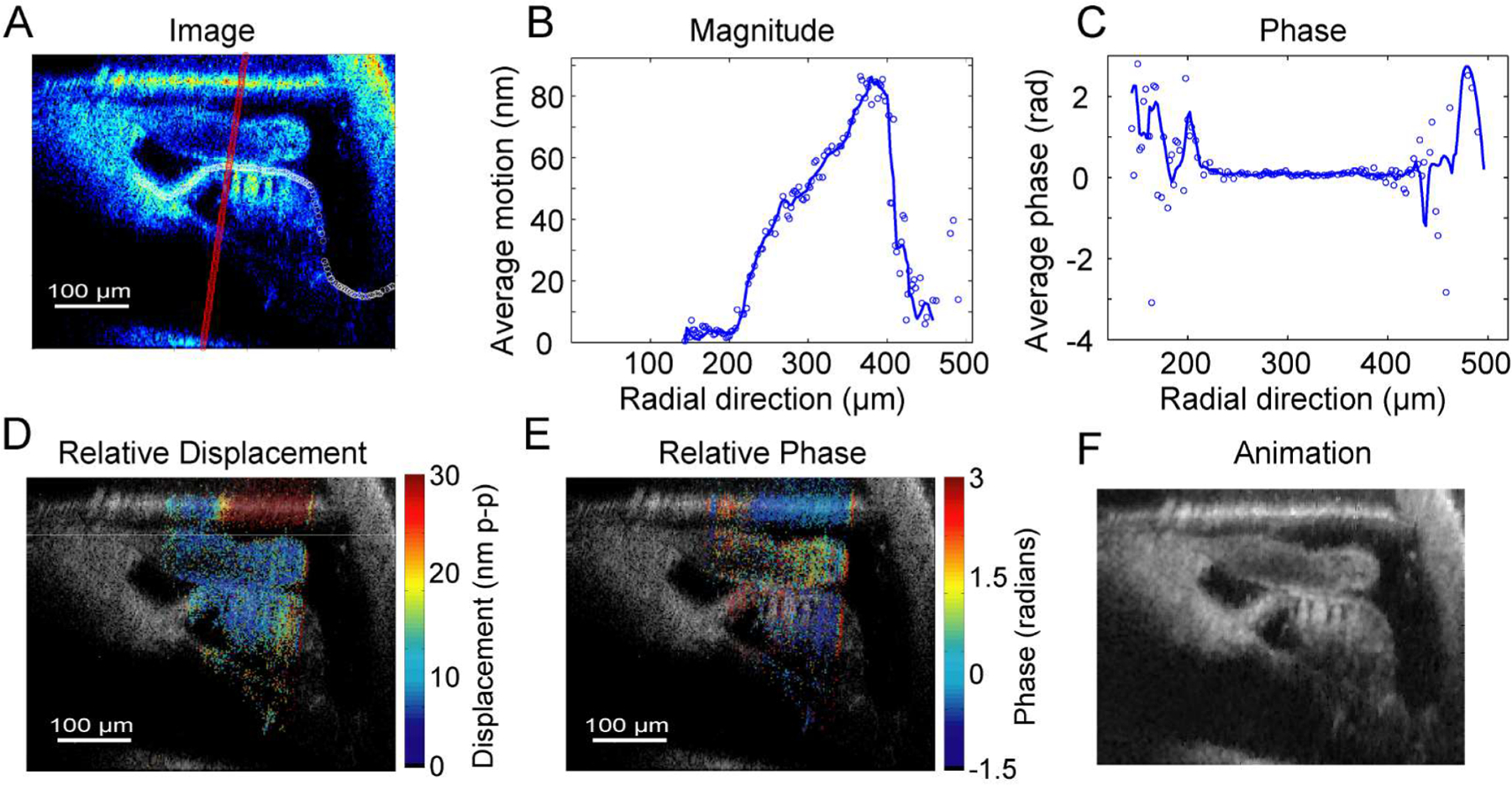
(A) Differential complex axial motion, referenced to the average complex motion along the reticular lamina (white circles), is determined by subtracting that motion along lines that parallel the outer pillar cells (red line). (B) Magnitude and (C) phase of reticular lamina motion along the reference line (white circles from (A)). (D) Relative displacement and (E) phase of the cochlear partition, referenced to reticular lamina. (F) First frame of an animation (see associated video in Supplementary Materials Section) of 150x amplified axial differential motion referenced to the RL (white circles in (A)).

This procedure is illustrated in Fig. 7. White circles indicate points on the reticular lamina that establish the desired reference frame (Fig. 7A). Fig. 7(B) and (C) are the measured magnitude and phase of displacement along that line of white circles, with a 6-point moving average shown as a solid blue line. The red line in Fig. 7(A) denotes the physiologically relevant analysis axis, and is chosen in this case to be parallel to the axis of the OHCs. The complex displacement of each point along the reference plane is subtracted from those along the analysis axis (parallel to the red line). Fig. 7(D) and (E) show the resulting magnitude and phase of the entire cochlear partition, in the frame of reference of the now stationary reticular lamina.

Fig. 7(F) shows the first frame of an animation (see associated video in Supplementary Materials Section) of the amplified (150x) relative motion referenced to the reticular lamina. The reticular lamina remains in the stationary frame of reference, while the rest of the structures are allowed to move. From this, behaviors such as an inflection of the basilar membrane arcuate and pectinate zones can be observed. The most significant relative motions are at the basilar membrane, which exhibits a rocking motion, however, it is difficult to separate outer hair cell (OHC) and Deiter’s Cell contributions. Future studies of longitudinally separated cochlear partition behavior could perhaps resolve the contributions of each.

## 4. Conclusion

*In vivo* studies of motions in the cochlear apex (Gao et al 2013, 2014; Recio-Spinoso and Oghalai, 2017, 2018; Dong et al, 2018; Meenderink et al, 2022a; Burwood et al, 2022; Meenderink and Dong 2022b, 2024) utilized low numerical aperture, long working distance objectives alongside spectral detection methods to effectively simultaneously scan the entire depth of focus of the objective with overlapping coherence gates. This greatly decreases scanning time (Bail et al, 1996) and provides longer working distances that are necessary for apical *in vivo* measurements, at the cost of decreased lateral resolution. In contrast, *ex vivo* studies allow for measurements of cochlear motions with higher lateral resolution, given the smaller working distances required. Although this comes with the additional disadvantage of requiring artificial sound stimulation, it has the advantage of being able to see structures moving and interacting with amazing detail. A recent excised *in vitro* study (Lin et al, 2024), looked at mechanical and electrical displacements of a single turn in the gerbil apex cochlear partition with impressive resolution in two axes: axial and radial.

Our study aims to preserve the structure of the excised cochlea, *in situ*, to measure absolute and relative motions of the cochlear partition with sound-like stapes-induced stimulation. To maximize resolution, we imaged the organ of Corti through a small hole, which we were unable to seal because of the small working distance of our high NA objective. This hole can increase the effects of the “fast wave” on our measurements, however, since we performed these measurements at the characteristic frequency, we believe the effect of the “fast wave” to be minimal (Dong and Cooper, 2006).

In conclusion, we have imaged and measured the response of the apical *in situ* cochlear partition in response to stapes-induced stimuli, at high resolution. We animated this motion in an absolute and relative frame of reference, to visualize the behavior of the cochlear partition under acoustic-like stimuli.

## Supplementary Material

Video 1 - Absolute Motion

Video 2 - Differential Motion

Supplementary material associated with this article can be found, in the online version, at doi:10.1016/j.heares.2025.109311.

## Data Availability

Data will be made available on request.

## Abbreviations:

OCT: Optical Coherence Tomography
DOCM: Doppler Optical Coherence Microscopy
DOCT: Doppler Optical Coherence Tomography
